# Attitudes and Practices Regarding Helicobacter Pylori Infection Among the Public in Jordan: A Cross-Sectional Survey

**DOI:** 10.7759/cureus.55018

**Published:** 2024-02-27

**Authors:** Nader Alaridah, Rayan M. Joudeh, Raba’a F. Jarrar, Assem AlRefaei, Nour Shewaikani, Hasan Nassr, Mohammad Jum’ah, Mallak Aljarawen, Haneen Al-Abdallat, Laith M. Haj-Ahmad, Murad T. Attal, Laith Hamdan Mansour, Mohammad A. AL-Foqaha'a, Muhannad M. Mahmoud, Anas H. A. Abu-Humaidan

**Affiliations:** 1 Department of Pathology, Microbiology and Forensic Medicine, School of Medicine, The University of Jordan, Amman, JOR; 2 College of Medicine, Sulaiman Alrajhi University, Al-Bukayriyah, SAU; 3 School of Medicine, The University of Jordan, Amman, JOR; 4 Faculty of Medicine, Al-Balqa Applied University, As-Salt, JOR

**Keywords:** general population, jordan, practice, attitude, helicobacter pylori

## Abstract

Background: Helicobacter pylori is a major infection that can cause a variety of complications, including stomach cancer and peptic ulcers. There is a scarcity of research on the awareness of H. pylori in the general population in Jordan. Because public awareness and behavioral changes are powerful tools in curbing transmission rates, this study evaluated Jordanians' beliefs and behaviors about H. pylori infection.

Methods: The study was carried out in 2021 between May and July. Those who met the requirements for inclusion were asked to fill out a questionnaire through interviews. The questionnaire had three sections: sociodemographic data, participants’ attitudes regarding H. pylori infection, and daily practices that could affect H. pylori transmission.

Results: Responses were collected from 767 participants, 50.7% were females, 65.8% were married, and 65.1% had a high educational level. Only 31.6% of the participants held a positive attitude. The female gender was significantly associated with better attitudes regarding H. pylori infection. One-third of the interviewed participants showed good practices. The female gender and being 50 years old and above were significantly associated with better practices.

Conclusion: This study demonstrated that attitudes and practices regarding H. pylori infection in Jordan were unsatisfactory. Subsequently, public health efforts should be aimed at modifying those behaviors to decrease the disease burden.

## Introduction

Based on a systematic review, 4.4 billion individuals tested positive for Helicobacter pylori in 2015 worldwide. The highest prevalence was in Africa, Latin America, the Caribbean, and Asia [1]. In many developing countries, H. pylori prevalence exceeds 80% among middle-aged patients [2]. In Jordan, one cross-national study found that H. pylori seroprevalence was approximately 88% of the population [3]. Among the socioeconomic variables that impact H. pylori prevalence include gender, age, employment, and alcohol usage [4]. In addition, a lack of sanitation, overpopulation, and polluted water sources may explain the increased prevalence rate of H. pylori in underdeveloped nations. Thus, improvements in hygiene and living conditions are significant factors in decreasing the infection prevalence [5-7]. Transmission routes of H. pylori are still unclear [8], but oral-oral, fecal-oral, or gastro-oral routes are suggested to be the most likely routes of transmission [9]. In many countries with high H. pylori infection rates, screening and eradication therapy rates are not satisfactory [10], which may be attributed to both low public awareness and the lack of effective healthcare systems. Peptic ulcers can occur as a result of H. pylori infection [11], and chronic infection of H. pylori is considered the main cause of developing non-cardia gastric cancer [12]. Despite the global decline of gastric cancer incidence over the past few years [13], it is responsible for over 750,000 deaths in 2020. As a result, stomach cancer ranked fifth in terms of cancer incidence and fourth in terms of cancer fatality rates globally [14]. There is a lack of recent epidemiological data about stomach cancer in Jordan; however, in Jordan, the Ministry of Health reported that stomach cancer accounted for 2.7% of all newly diagnosed cancer cases in 2012 and was the sixth leading cause of cancer-related fatalities [15].

Few studies have investigated the attitude level in the general population regarding H. pylori infection. A study done in the United Arab Emirates (UAE) showed that 39% of people who had symptomatic H. pylori did not go to a doctor due to their belief in herbal treatments, as well as the avoidance of seeking medical care due to the high healthcare expenses [16]. Even though 41% of participants in another Chinese research had an H. pylori infection, 86% of the subjects believed they were not infected [17]. The attitudes and practices regarding H. pylori are influenced by some sociodemographic factors [16,18,19]. Tackling H. pylori transmission in society, along with providing an effective eradication therapy, is crucial in decreasing morbidity and mortality associated with the infection [20]. Accordingly, the high seroprevalence of H. pylori and the lack of reports on the current situation among the Jordanian public pose significant problems to public health that threaten the provision of effective treatment [3,21]. Thus, the primary objective of the current research is to investigate public attitudes and practices about H. pylori infection in Jordan, as well as investigate the sociodemographic predictors associated with a positive attitude and better behavioral practices.

## Materials and methods

Sample recruitment and study setting

A cross-sectional interview-based study was carried out with patients who visited Jordan University Hospital's outpatient clinics in 2021 between May and July. Students from 4th and 5th medical colleges were trained by the research team, to interview the participants. They were selected from outpatient clinics by using the convenience sampling method, in which each student was assigned to have an interview with random patients who visited the outpatient clinics at Jordan University Hospital. Additionally, each student was assigned to a specific outpatient clinic, to avoid repeated responses from patients. After patients agreed to participate, they were provided complete information about the study and its intended goals. Age above 18, ability to converse verbally in Arabic, and willingness to engage in the study were the inclusion criteria. Participants who had never heard of H. pylori and did not meet the eligibility requirements were excluded. In addition, repeated responses were removed. The study requires a minimum sample size of 385 people, which was estimated using a 5% margin of error and a 50% prevalence [22].

Development of the survey

**Figure 1:**
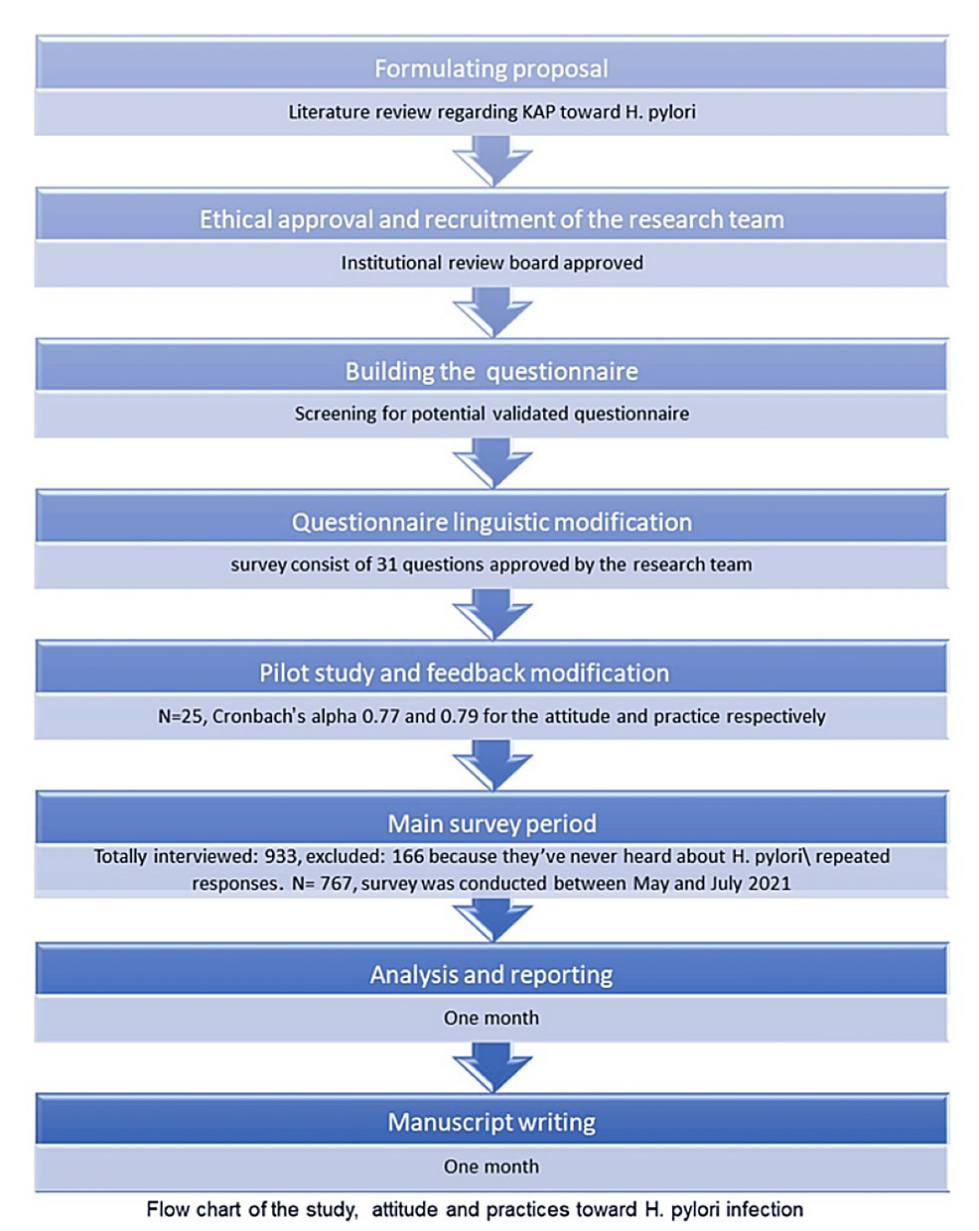
Flow chart of the study, attitude, and practice regarding H. pylori infection

Since there are no validated questionnaires to evaluate the attitudes and practices regarding H. pylori among the general population, we developed a survey based on literature reviews [23] and other studies [16,19,24]. A flow chart of the study processes is presented in Figure 1. The questionnaire was produced in English and then translated into the local spoken language (Arabic) by competent speakers of both languages. It was then refined to be understandable to the general public. The questionnaire was assessed and finalized by a gastroenterology specialist. The questionnaire was pilot-studied on 25 participants, and slight modifications were made to the language of some questions. The internal consistency was assessed using Cronbach’s alpha 0.77 and 0.79 for the attitude and practice parts, respectively.

Measurement tool

The questionnaire consisted of 31 questions, divided into three main parts: sociodemographic, attitudes, and practices regarding H. pylori infection. The sociodemographic data included gender, age, educational level, marital status, occupational position, residential location, and prior awareness of H. pylori. The attitude part consisted of 15 items covering three aspects: treatment, screening, and prevention. The practice part consisted of eight items covering three aspects: eating and drinking habits, hygiene, and antibiotic use. The attitude score assessment was measured on a 3-point Likert scale with the following options per question: agree, disagree, or maybe. Moreover, the practice score assessment was based on a 5-point Likert scale having the following options for each question: always, usually, sometimes, often, and never. We used a 75% bloom cutoff to classify participants' attitudes and practices into the binary category. This cutoff value is also based on previously published knowledge, attitudes, and practices (KAP) studies [25,26].

Ethical considerations

The Institutional Review Board (IRB) of the University of Jordan, Amman, Hashemite Kingdom of Jordan (reference number: 10/ 2021/ 27975), examined and approved the study protocol in meeting No. 2021/18. It was written in accordance with the Helsinki Declaration's principles. Prior to the start of filling out the questionnaire, informed consent was obtained from all candidates. After that, all the data was collected and classified. They were retained, and only the principal investigator could access them.

Statistical analysis

Data were gathered and imported into Microsoft Excel (2016) before being transferred into Statistical Product and Service Solutions (SPSS, version 25; IBM Corp., Armonk, New York) for analysis. Descriptive statistical methods were applied to present quantitative and categorical data, and they were reported as percentages and frequencies or mean and standard deviation (SD). The chi-square test was used to examine the relationships between demographic characteristics and attitudes and practices (at the binary level). Merely statistically significant associated variables were analyzed in the multivariate logistic regression analysis, which was adjusted for possible confounders to determine each variable's independent impact. To establish the statistical significance of the provided data, a p-value of 0.05 and a 95% confidence interval were used.

## Results

Sociodemographic characteristics of the participants

A total of 767 participants completed the surveys. Females made up around half of the sample (50.7%). A total of 505 (65.8%) of all responders were married, 499 (65.1%) of the participants had a high level of education (had a diploma degree or higher), and 111 (14.5%) of the participants worked in the medical field. In terms of residence place, 678 (88.4%) of all participants lived in cities. The summary of sociodemographics is shown in Table 1.

**Table 1:** Sociodemographic characteristics of H. pylori questionnaire participants *Education level: High education means if the participant has a diploma of any higher degrees

Characteristics	Number (n=767)	Percentage (%)
Age		
18-29	229	29.9
30-39	131	17.1
40-49	163	21.3
50 and above	244	31.8
Gender		
Male	378	49.3
Female	389	50.7
Education Level*		
Low Education	268	34.9
High Education	499	65.1
Marital status		
Married	505	65.8
Unmarried	262	34.2
Job Field		
Medical	111	14.5
Non-medical	387	50.5
No work	269	35.1
Residence place		
Urban	678	88.4
Rural	89	11.6

Attitudes regarding H. pylori and its associated factor

Table 2 shows a summary of the participants' correct responses to the attitude questions. When the participants scored 12 points or more, they were considered to have good attitudes; when they scored less than 12 points, they were considered to have poor attitudes.

**Table 2:** Participants’ attitudes regarding H. pylori

Topic	Correct answer	Correct answer, n (%)
Seeking medical advice if you have any symptoms of H. pylori infection	Agree	723 (94.3)
Recommending family members to seek medical advice if they noticed any symptoms of H. pylori infection	Agree	740 (96.5)
Considering screening for H. pylori infection	Agree	211 (27.5)
Completing the whole course of treatment, even if no improvement is noticed at the start	Agree	637 (83.1)
Visiting the same physician for follow-up if there is no improvement	Agree	430 (56.1)
Special diet can improve the symptoms	Agree	599 (78.1)
Believe that herbal medicine can eradicate H. pylori	Disagree	351 (45.8)
Believe that someone can get reinfected after eradication	Agree	606 (79.0)
Regular hand washing could decrease the chance of H. pylori infection	Agree	655 (85.4)
Drinking filtered/bottled water could decrease the chance of H. pylori infection	Agree	601 (78.4)
Eating fast food could increase the risk of H. pylori infection	Agree	684 (89.2)
Living with someone infected with H. pylori could increase the risk of H. pylori infection	Agree	282 (36.8)
Living in crowded places could increase the risk of H. pylori infection	Agree	413 (53.8)
H. pylori infection can be treated easily	Disagree	204 (26.6)
Prompting awareness toward H. pylori infection could help in the treatment and prevention of health consequences	Agree	736 (96.0)

Only 31.6% of the participants had a good attitude. In terms of attitudes about seeking medical advice and screening for H. pylori, 94.3% and 96.5% of the participants said that they would seek medical advice for themselves and would propose it to their family and friends if they had symptoms of H. pylori infection, respectively. On the other hand, only 27.5% of the participants would go for screening for H. pylori infection. For the treatment adherence and follow-up, 83.1% of the participants would agree to complete the whole course of the treatment assigned to them. Even if no improvement was noticed, 56.1% of the participants would return to the same physician for a follow-up. Regarding their beliefs about herbal treatment and the role of diet on H. pylori infection, 78.1% of the participants believed that some special diet would help in improving symptoms of H. pylori infection, and only 45.8% of them believed that herbal treatment has no role in H. pylori eradication. As for the attitudes about H. pylori prevention, 85.4% of participants agreed that regular handwashing would prevent H. pylori infection, 78.4% believed that drinking treated water would help in prevention, and 89.2% believed that eating fast food would increase the infection risk. Additionally, 36.8% and 53.8% of participants believed that close contact with someone infected by H. pylori and living in crowded places would increase the risk of getting the infection, respectively. Only 26.6% of the population declared that H. pylori cannot be easily treated, while 96% of the population agreed that promoting awareness about H. pylori would help in treating it and prevent its health consequences. When assessing variables associated with attitudes, only gender showed a significant association (Table 3). Nonetheless, age, job field, marital status, education, and residence place showed no significant association with the level of attitude in the chi-square test (Table 4). Hence, these variables were not included in the logistic regression analysis. Univariate logistic regression analysis showed a significant association with attitudes score, as shown in Table 5: gender (female: OR = 1. 577, CI = 1.159-2.144, p = 0.004; ref: male).

**Table 3:** Association between sociodemographic characteristics of general population, attitudes, and practices regarding H. pylori (n = 767) * Those who scored 12 or above in all the items in Table 2 were considered to have a good attitude toward H. pylori infection ** Those who scored 5 or above in all the items in Table 5 were considered to have good practice against H. pylori infection *** P-value by the chi-square test

	Level of Attitude*				Level of Practice**				
	Good (243)	Bad (524)		P-value	Good (247)			Bad (520)	P-value***
Age				0.994					0.002
18-29	72		157		64		165		
30-39	41		90		30	101			
40-49	51		122		55	108			
50 and above	79		165		98	146			
Gender				0.004					0.015
Female	142		247		141	248			
Male	101		277		106	272			
Marital status				0.414					0.823
Married	155		350		164	341			
Unmarried	88		174		83	179			
Education level				0.988					0.017
Lower education	85		183		101	167			
Higher education	158		341		146	353			
Job field				0.068					0.011
Medical	44		67		25	86			
Non-medical	110		277		120	267			
No work	89		180		102	167			
Living conditions				0.357					0.108
Urban	211		467		225	453			
Rural	32		57		22	67			

**Table 4:** Logistic regression analysis establishing the associations between attitude and practice among the general population in Jordan * P-value by univariate logistic regression; ** P-value by multivariate logistic regression; *** Those variables labelled as “NA” were not statistically significant in the chi-square test in Table 3. Hence, they were not included in this logistic regression. Abbreviations: OR, Odds Ratio; CL, Confidence Interval, NA, Not Applicable

Covariates	Attitude			Practice		
	OR	CI	P-value	OR	CI	P-value
Age						
18-29	NA***			Reference		
30-39				0.674	0.459-1.274	.303
40-49				1.148	0.730-1.870	0.550
50 and above				1.575	1.053-2.346	0.027**
Gender:						
Male	Reference			Reference		
Female	1.577	1.159-2.144	0.004*	1.420	1.019-1.981	0.039**
Education level:	NA***					
Lower education				1.225	0.860-1.746	0.261
Higher education				Reference		
Job field:	NA***					
Medical				0.664	0.379-1.164	0.153
Non-medical				0.949	0.647-1.393	0.791
No work				Reference		

**Table 5:** Participants’ practices toward H. pylori infection

Topic	Correct answer	Correct answer, n (%)
Drinking filtered water	Always	622 (81.1)
Drinking alcohol	Never	725 (94.5)
Using public restrooms	Never	150 (19.6)
Eating fast food on a regular basis (>4 times a week)	Never	73 (9.5)
Washing vegetables/fruits before eating them	Always	629 (82.0)
Using antibiotics without a prescription	Never	365 (47.6)
Sharing drinking cups	Never	432 (56.3)
Sharing food utensils	Never	581 (75.7)

Practices regarding H. pylori and its associated factor

The summary of participants’ answers to the practice questions is shown in Table 5. Participants who scored 5 or more points were reported to have good practice, while those who scored less than 5 points were reported to have bad practice. Approximately, only one-third (31.6%) of the participants had good practice, 81.1% of participants reported that they always drink filtered water, and 94.5% reported they had never drunk alcohol. Additionally, 19.6% and 9.5% of the participants reported that they never use public bathrooms and do not eat fast food on a regular basis, respectively.

When the participants were asked about using antibiotics without a prescription, 47.6% of them never consumed antibiotics without a prescription. Additionally, 82% of the participants reported that they wash fruits and vegetables always before eating them. When participants were asked about sharing cups and utensils, 56.3% and 75.7% reported that they never shared cups and utensils with others, respectively. Statistically significant associations were noticed between sociodemographic data and practices, as shown in Table 3. On multivariate logistic regression analysis, significant associations were found between practice and the following: age (50 and above: OR = 1.575, CI = 1.053-2.346, p < 0.027, ref: 18-29) and gender (female: OR = 1.420, CI = 1.053-2.346, p = 0.039; ref: male). Other sociodemographic data such as marital status, residence place, and other age groups were not significantly associated with practice (Table 3), so they were not included in the multivariate logistic regression analysis.

## Discussion

With over 700 participants, this study provided insight into the attitudes and practices regarding H. pylori infection among Jordanians. The interview-based questionnaire was performed at Jordan University Hospital, which is one of the largest tertiary hospitals in the capital Amman, and serves patients from various geographical regions in Jordan. In addition, the demographics of the participants revealed that different age groups, genders, and, to a lesser extent, educational levels were represented equally in this study. This highlights the demographics associated with better practices and attitudes, aiming to assist public health policies set to decrease the disease burden of H. pylori infection. In terms of health-seeking behavior, the willingness to seek medical advice was high in the tested population, in contrast to a recent study done in the UAE that indicated unsatisfactory levels of medical advice-seeking behavior in case of infection [16]. However, only 27.5% of the participants agreed to have a screening test for H. pylori, which is comparable to the UAE study [16]. The majority of our survey participants thought that increasing H. pylori knowledge would help with the treatment and prevention of H. pylori infection.

In general, around one-third of the participants had a good H. pylori practice. We observed that being female is highly related to a more positive attitude. Being older than 50 and being female were also predictors of better practice. Besides biological, social, and cultural factors, females might have more tendency for hygienic attitudes and engage in healthy behaviors associated with primary prevention. Furthermore, older adults may have better practices toward infecting agents due to their experience, health concerns, and sense of social responsibility. Three-quarters of participants always drink purified water and wash their vegetables and fruits. A considerable proportion of the population regularly use public restrooms and eat fast food. These activities are critical in terms of H. pylori transmission, particularly in developing and underdeveloped countries [16,24]. Many studies indicated the presence of H. pylori genetic material in drinking water and some food types (e.g., meat, fruits, vegetables, and unclean items) [27,28]. Because person-to-person contact is thought to be the most likely mode of transmission, various sociocultural practices have been linked to an increase in the incidence of H. pylori [29]. A substantial proportion of our population admitted to sharing utensils and drinking cups with their family. This might account for the high seroprevalence of H. pylori (88.6%) in Jordan [3].

Unsurprisingly, more than half of respondents reported that they use antibiotics without a doctor's prescription, a behavior that had been reported previously among the Jordanian public and even among pharmacists [30]. Limited understanding of antibiotic usage and the rise in antibiotic resistance go hand in hand, both result in a significant increase in disease burden [31,32]. In addition, there are concerns about how much Jordanian gastroenterologists follow the international recommendations for identifying and treating H. pylori infection and whether they are aware of the local antibiotic resistance to the standard therapeutic regimens. However, these are challenging topics of interest that require further research in Jordan. A comparable study conducted in Italy found that Italian gastroenterologists adhere to international guidelines for the management of H. pylori infection [33]. The current study demonstrated unsatisfactory results regarding attitudes and practices among the Jordanian population, which is inconsistent with other studies done in the UAE and China [16,24], highlighting the pervasiveness of this global problem and the need to address it. Several measures could be implemented to enhance attitudes and practices regarding H. pylori infection and its health-related consequences. For example, conducting regular educational campaigns at hospitals and primary care clinics, with an emphasis on behaviors that could increase H. pylori transmission. Additionally, healthcare providers should be encouraged to counsel their patients on the proper behaviors related to H. pylori transmission and treatment. On the contrary to other studies [34-36], an unproportionate geographical representation of participants, who lived in rural areas (11.6%), might have contributed to the insignificance of residence place. Furthermore, neither the participants' degree of education nor their work field showed any statistically significant correlation with their attitude regarding H. pylori infection. Future research should closely consider those key demographic parameters to confirm or refute the findings and their generalizability beyond the targeted population. Finally, data on the prevalence of H. pylori, its antibiotic resistance patterns, and complications of infection should all be monitored through a dedicated national registry, which will help guide future policies and underscore the extent of the problem in society.

## Conclusions

This study showed that attitudes and practices regarding H. pylori infection among the Jordanian public need to be improved. We found that being a female was strongly associated with a better attitude, while being a female and being older than 50 years old were associated with better practices. Given the high prevalence of infection in society, urgent measures should be implemented to address behaviors that increase transmission of the pathogen such as sharing utensils and drinking cups, as well as behaviors that could lead to treatment failure, such as the misuse of antibiotics.
